# Prognostic significance of early alpha fetoprotein and des-gamma carboxy prothrombin responses in unresectable hepatocellular carcinoma patients undergoing triple combination therapy

**DOI:** 10.3389/fimmu.2024.1508028

**Published:** 2024-12-12

**Authors:** Teng Zhang, Wengang Li, Qian Chen, Weiping He, Jing Sun, Dong Li, Quan Wang, Xuezhang Duan

**Affiliations:** ^1^ Department of Radiation Oncology, Senior Department of Oncology, The Fifth Medical Center of PLA General Hospital, Beijing, China; ^2^ Department of Oncology, The 983rd Hospital of Joint Logistic Support Force of PLA, Tianjin, China; ^3^ Medical School of Chinese PLA, Beijing, China

**Keywords:** hepatocellular carcinoma, triple combination therapy, stereotactic body radiation therapy (SBRT), alpha fetoprotein (AFP), des-gamma-carboxy prothrombin (DCP)

## Abstract

**Background:**

Recent advancements in combination therapy for unresectable hepatocellular carcinoma (uHCC) have shown promise, but reliable serological prognostic indicators are currently lacking for patients undergoing triple combination therapy of stereotactic body radiation therapy (SBRT), immunotherapy, and targeted therapy. We aimed to investigate the prognostic significance of early alpha fetoprotein (AFP) and des-gamma-carboxy prothrombin (DCP) responses in these patients.

**Methods:**

This retrospective research included 115 uHCC patients treated with SBRT in combination with immunotherapy and targeted therapy (triple therapy) at our institution from April 2021 to December 2022. Participants were categorized into high AFP and high DCP cohorts based on baseline levels. AFP and DCP responses were defined as decreases from baseline of over 50% and 70%, respectively, according to ROC curve analysis. Differences in overall survival (OS), progression-free survival (PFS), and objective response rate (ORR) were assessed between the tumor biomarker response and non-response groups.

**Results:**

Multivariate analysis indicated that AFP or DCP response at 6-8 weeks post-therapy significantly influenced ORR (high AFP cohort: odds ratio [OR] 5.50, 95% CI 2.04-14.83, p=0.001; high DCP cohort: OR 7.99, 95%CI 2.82-22.60, p<0.001). The median PFS was notably longer in tumor biomarker response groups (high AFP cohort: 13.7 vs 6.2 months, hazard ratio [HR] 0.36, 95% CI 0.20-0.62, p<0.001; high DCP cohort: 15.6 vs 9.3 months, HR 0.44, 95% CI 0.26-0.74, p=0.002). AFP or DCP response was associated with prolonged OS (high AFP cohort: not reached vs. 21.9 months, HR 0.47, 95% CI 0.22-0.99, p=0.047; high DCP cohort: not reached vs. 20.6 months, HR 0.35, 95% CI 0.14-0.86, p=0.022).

**Conclusion:**

AFP or DCP response at 6-8 weeks post-therapy predicts better oncological outcomes in patients with uHCC treated with triple therapy.

## 1 Introduction

Hepatocellular carcinoma (HCC) remains a major global health concern, representing the sixth most prevalent tumor and the third leading cause of cancer-related deaths globally (1, 2). Around 80% of HCC cases are diagnosed at advanced stages, with a discouraging 5-year survival rate of approximately 18% (3, 4).

Although various combination therapies based on immune checkpoint inhibitors (ICIs) and targeted agents, including atezolizumab plus bevacizumab (5), sintilimab combined with a bevacizumab biosimilar (IBI305) (6), camrelizumab plus rivoceranib (7), have become the preferred option for the systemic therapy of unresectable HCC (uHCC), the objective response rate (ORR) remains unsatisfactory (8, 9). Investigations are underway into novel therapeutic approaches for HCC and a spectrum of other malignancies, aiming to enhance treatment efficacy and patient outcomes (10–12). The increasing utilization of radiological technology has led to the growing recognition of stereotactic body radiation therapy (SBRT) as a significant treatment approach for HCC (13, 14). Furthermore, the synergistic anti-tumor effects stemming from multiple mechanisms in this triple therapy contribute to an enhanced anti-tumor activity, leading to improved prognosis (15–17). Indeed, despite the striking efficacy of triple therapy, no biomarkers have been currently validated helpful to identify patients who can respond to this treatment.

At present, there is no ideal surrogate endpoint for overall survival (OS) in HCC (1). ORR and progression-free survival (PFS) have been used as surrogate endpoints in clinical trials, but their correlation with OS is limited (18, 19). It highlights the need for more accurate substitutes that could reflect OS better and earlier and serve as reliable indicators of treatment effectiveness. Alpha-fetoprotein (AFP) and des-gamma-carboxy prothrombin (DCP) are commonly tested serum biomarkers for surveillance of high-risk individuals in HCC (20, 21). Moreover, their dynamic changes have been extensively studied as prognostic biomarker for response to systemic or some locoregional treatment such as transarterial chemoembolization (TACE) in HCC (22–25). However, there is a lack of studies using tumor biomarker response to predict radiotherapy efficacy. In addition, the cut-off values of biomarker response are often determined arbitrarily, which can introduce potential bias and affect the accuracy and reliability of the findings.

Therefore, we sought to explore the clinical value of AFP and DCP response as prognostic indicators in patients with uHCC undergoing triple therapy. The primary endpoint of this study was ORR by imaging assessment per modified RECIST (mRECIST) (26, 27), and the secondary endpoint was PFS and OS.

## 2 Materials and methods

### 2.1 Study design and patients

Overall, a cohort of 115 uHCC patients underwent triple therapy from April 2021 to December 2022 at our institution were included in this retrospective study. All patients were deemed unresectable through multidisciplinary evaluation. The following inclusion criteria were used: (1) no previously received SBRT, immunotherapy or targeted therapy, (2) Barcelona Clinic Liver Cancer (BCLC) stage B or C, (3) Eastern Cooperative Oncology Group (ECOG) performance status score of 0 or 1, (4) Child-Pugh classification A and B7, and at least 1 measurable intrahepatic lesion. The exclusion criteria were without imaging evaluation or incomplete data on tumor markers, combination of PD-1 inhibitors less than 2 cycles and no elevation of both AFP and DCP at baseline. In accordance with ethical standards, this study strictly adhered to the principles stated in the Declaration of Helsinki. Ethical approval was obtained from the Ethics Committee of the PLA General Hospital’s Fifth Medical Centre. Informed consent was unnecessary for participation due to the retrospective nature.

### 2.2 Treatment protocol

After the implantation of 3-5 localization markers, patients underwent SBRT using a CyberKnife VSI image-guided robotic stereotactic radiosurgery system. Following CT simulation to identify the treatment site, an oncologist contoured the gross tumor volume (GTV) and delineated the organs at risk (OARS). The GTV included the tumor and portal vein tumor thrombus (PVTT) if it met the dose-volume limits of the critical organs. Otherwise, just the PVTT is described as the GTV (28). The planning target volume (PTV) extends the GTV by 3-5 mm and avoided the OARs. The prescribed doses were 45 to 55 Gy/5 to 10 fractions. Acceptable doses of OARs were derived from the American Association of Physicists in Medicine (AAPM) TG-101 report (29).

All patients were initiated ICIs in combination with targeted drugs within one week after the completion of the last radiotherapy session. The specific regimens included Sintilimab + Bevacizumab, Sintilimab + Lenvatinib, Tislelizumab + Lenvatinib, or Toripalimab + Lenvatinib. The prescribed dosages were as follows: Sintilimab at 200mg every three weeks, Tislelizumab at 200mg every three weeks, Toripalimab at 240mg every three weeks, and the recommended initial dose of lenvatinib for is 12 mg/day for individuals weighing more than 60 kg, and 8 mg/day for those weighing 60 kg or less. Bevacizumab was administered at 15 mg/kg of body weight every three weeks.

### 2.3 Data collection and follow-up

We systematically gathered and analyzed baseline clinical parameters and follow-up data for applicable individuals, encompassing age, gender, etiology, ALBI classification (30), BCLC stage, blood count, transaminase, AFP, DCP, the occurrence of macrovascular invasion and extrahepatic metastases. Baseline AFP and DCP levels were assessed within one week prior to the start of triple therapy. High AFP was defined >10 ng/ml and high DCP was defined >40 mAU/ml according to our institution’s reference range. To monitor AFP and DCP responses, data were obtained 6-8 weeks after the triple therapy. Radiological response was evaluated using Magnetic Resonance Imaging (MRI) per modified Response Evaluation Criteria in Solid Tumors (mRECIST) (26, 27), with tumor assessments conducted every 6-8 weeks by three expert radiologists. ORR was determined on the basis of optimal treatment response, with complete remission (CR) or partial response (PR) lasting≧1 month. Patients received triple therapy until the onset of intolerable adverse events or disease progression. PFS was defined as the duration between the initiation of treatment and the occurrence of disease progression, death, or the final follow-up. OS was the time between the commencement of therapy and death or the last follow-up.

### 2.4 Statistical analysis

Continuous data were analyzed using independent T-tests and reported as the median and interquartile spacing. Numbers and percentages were used to characterize the categorical data, and chi-square or Fisher exact tests were applied to compare the results. ROC curves were then plotted between the radiographic response and the magnitude of tumor marker decline at 6-8 weeks post-therapy. The biomaker cut-offs were chosen by identifying the points on the ROC curve that maximized the sum of sensitivity and specificity in differentiating patients by best confirmed response. To find acceptable objective response (CR+PR) predictors, binary logistic regression analysis was employed. The Kaplan-Meier approach produced survival curves, and the log-rank test was used to assess group differences. Cox regression models were used to identify PFS and OS predictors. In the multivariate analysis, factors with p < 0.1 in the univariate analysis were included, and p < 0.05 was deemed statistically significant. Software called SPSS and Graphpad Prism were used for all statistical analyses.

## 3 Results

### 3.1 Patient description

**Figure 1:**
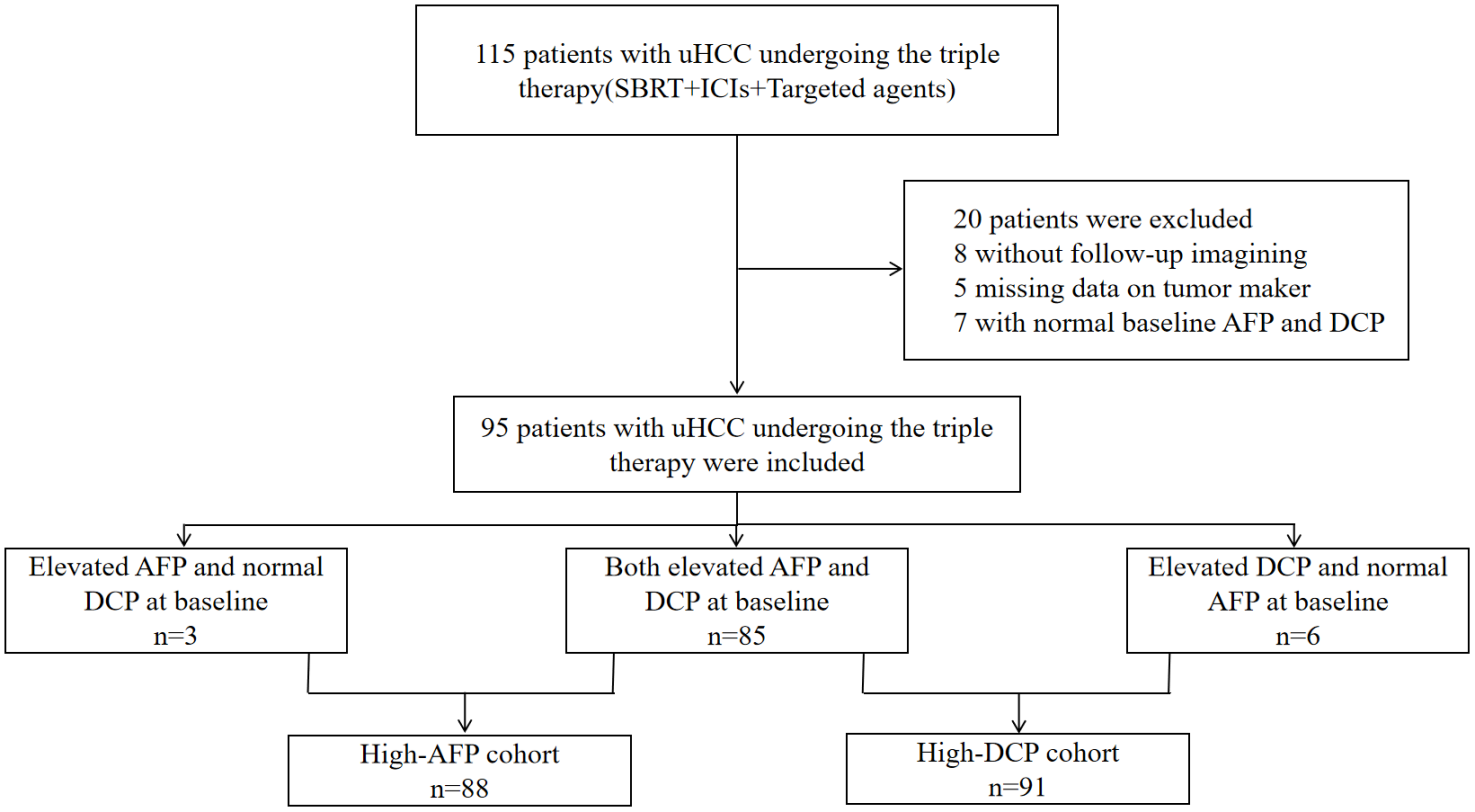
Flow diagram of the research design.

A total of 20 of the 115 patients were excluded due to no imaging evaluation (n=8) or missing tumor marker data (n=5), or no elevated baseline AFP and DCP levels (n=7) (**Figure 1**). The baseline characteristics of the remaining individuals were shown in **Table 1**. Patients were predominantly over 50 years of age (70, 73.7%) and male (88, 92.6%). Furthermore, a significant proportion of patients had a history of chronic hepatitis B infection (89, 93.7%). Vascular invasion was found in 75 (78.9%) patients and extrahepatic metastases were present in 18 (19.0%) patients. According to the BCLC staging, 9 patients (9.5%) were classified as stage B, and 86 patients (90.5%) were classified as stage C. In terms of Child-Pugh classification, 73 patients (76.8%) were classified as class A, while 22 patients (23.2%) fell into class B. Regarding the ALBI classification, 18 patients (19.0%) were assigned to class 1, and 77 patients (81.0%) to class 2. The median baseline AFP was 604.5 ng/ml, while the median baseline DCP was 1396.0 mAU/ml.

**Table 1:** Clinical characteristics of HCC patients receiving triple therapy (SBRT+ICIs+targeted agents).

Characteristics	Total	High-AFP	High-DCP
Sample size	95	88	91
Age, years			
≤50	25(26.3)	24(27.3)	25(27.5)
>50	70(73.7)	64(72.7)	66(72.5)
Chronic liver disease			
HBV	89(93.7)	83(94.3)	85(93.4)
Others	6(6.3)	5(5.7)	6(6.6)
Gender			
Male	88(92.6)	81(92.0)	84(92.3)
Female	7(7.4)	7(9.0)	7(7.7)
Vascular invasion			
No	20(21.1)	18(20.5)	20(22.0)
Yes	75(78.9)	70(79.5)	71(78.0)
Extrahepatic spread			
No	77(81.0)	71(80.7)	74(81.3)
Yes	18(19.0)	17(19.3)	17(18.6)
BCLC stage			
B	9(9.5)	8(9.1)	9(9.9)
C	86(90.5)	80(90.9)	82(90.1)
Child-pugh			
A	73(76.8)	66(75.0)	70(75.3)
B	22(23.2)	22(25.0)	21(24.7)
ALBI grade			
I	18(19.0)	16(18.2)	17(18.7)
II	77(81.0)	72(81.8)	74(81.3)
Platelet count (× 10^9^/L)	137(84-212)	136(84-205)	137(84-212)
ALT (U/L)	35.0(24.5-49.0)	35.5(24.8-50.8)	35.0(24.5-48.5)
AST (U/L)	50.0(33.0-82.5)	51.0(33.5-86.5)	50.0(33.0-82.5)
Neutrophil (× 10^9^/L)	2.8(1.9-4.3)	2.8(1.9-4.3)	2.8(1.9-4.3)
Baseline AFP level (ng/ml)	604.5(53.4-3806.0)	948.5(95.4-4296.2)	754.0(53.4-4048.5)
Baseline DCP level (mAU/ml)	1396.0(130.0-20312.0)	1934.0(130.0-21773.8)	2386.0(158.0-20886.5)
AFP reduction>50%	/	55(62.5)	/
DCP reduction>70%	/	/	42(46.1)
ORR	61(64.2)	55(62.5)	57(62.6)
Therapy			
SBRT+Sintilimab+Bevacizumab	45(47.4)	43(48.9)	43(47.3)
SBRT+Sintilimab+Lenvatinib	35(36.8)	30(34.1)	33(36.3)
SBRT+Tislelizumab+Lenvatinib	4(4.1)	4(4.5)	12(13.2)
SBRT+Toripalimab+Lenvatinib	11(12.5)	11(12.4)	3(3.2)

Values are expressed as numbers (%), median (interquartile range); HCC, hepatocellular carcinoma; HBV, hepatitis B virus; BCLC, Barcelona Clinic Liver Cancer staging; ALBI, albumin-bilirubin; ALT, alanine aminotransferase; AST, aspartate aminotransferase; AFP, alpha-fetoprotein; DCP, des-gamma-carboxyprothrombin; ORR, objective response rate; SBRT, stereotactic body radiation therapy; ICIs, immune checkpoint inhibitor.

### 3.2 Predictor of objective response in the high-AFP cohort

A total of 88 individuals exhibited elevated baseline AFP levels, 64 (72.7%) of them were older than 50, and 83 (94.3%) were infected with HBV. Of this subgroup, 70(79.5%) had vascular invasion, and 17 (19.3%) had extrahepatic metastases, and the median baseline AFP value was 948.5 ng/ml (**Table 1**).

**Figure 2:**
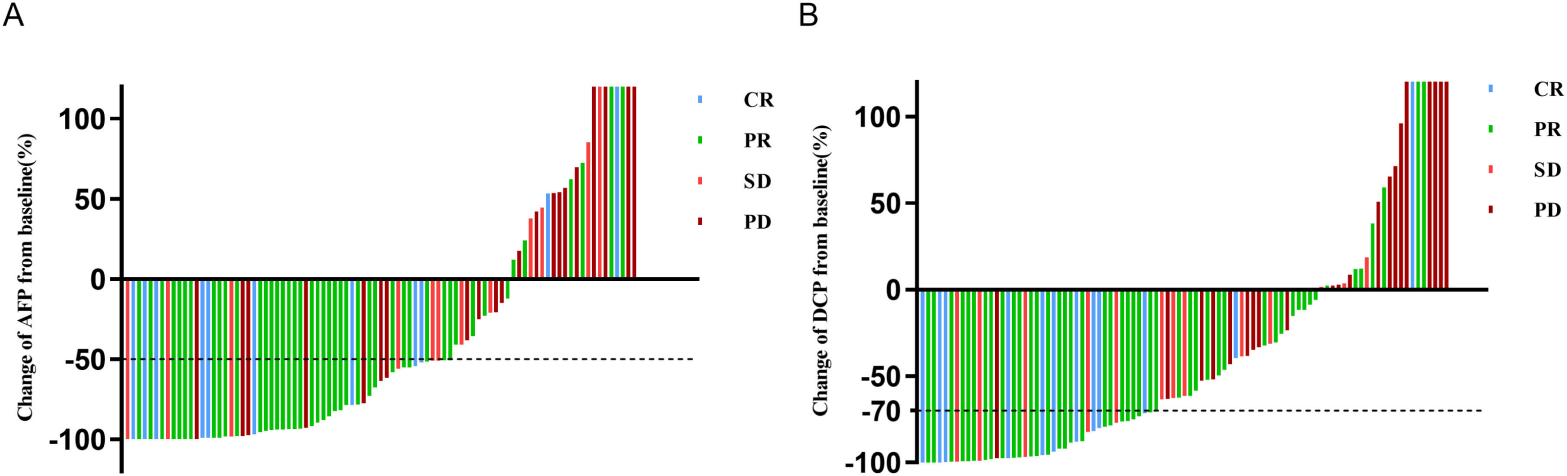
Waterfall plot of change in **(A)** AFP or **(B)** DCP at 6-8 weeks from baseline and best response per mRECIST. AFP, alpha-fetoprotein; DCP, des-gamma-carboxy prothrombin; mRECIST, modified response evaluation criteria in solid tumors; CR, complete response; PD, progressive disease; PR, partial response; SD, stable disease.

After undergoing triple therapy for 6–8 weeks, analysis based on the ROC curve revealed that a 51.5% decrease in AFP was the optimal threshold, corresponding to the maximum Youden index value of 0.414 (**Supplementary Table S1**). Consequently, a decrease in AFP of 50% was established as the cut-off value for defining AFP response (**Supplementary Table S1**). In this group, a total of 55 patients (62.5%) exhibited AFP response (**Table 1**). As depicted in the waterfall diagram in **Figure 2A**, most of the patients who demonstrated AFP response also achieved objective response. As shown in **Table 2**, the multivariate analysis confirmed that AFP response independently influenced objective response (OR 5.50, 95% CI 2.04-14.83; p=0.001).

**Table 2:** Univariate and multivariate analyses for factors associated with objective response in the high AFP cohort (n=88).

Variables	Univariate Analyses		Multivariate Analyses	
	OR (95%CI)	p	OR (95%CI)	p
Age, years (≤50/>50)	0.75(0.29-1.95)	0.553		
Gender (Female/Male)	3.92(0.45-34.09)	0.216	6.31(0.64-62.01)	0.114
Extrahepatic spread (No/Yes)	1.01(0.35-2.88)	0.986		
BCLC(B/C)	0.55(0.13-2.35)	0.418		
Child-Pugh (A/ B)	1.34(0.51-3.48)	0.553		
Tumor size (cm) (≤5/>5)	0.90(0.37-2.17)	0.810		
Baseline AFP (ng/ml)	1.00(1.00-1.00)	0.122	1.00(1.00-1.00)	0.421
ALBI grade (1/2)	0.96(0.31-2.93)	0.939		
AFP response (Yes/No)	4.97(1.95-12.66)	**0.001**	5.50(2.04-14.83)	**0.001**
Vascular invasion (No/Yes)	0.75(0.27-2.10)	0.580		

BCLC, Barcelona Clinic Liver Cancer staging; ALBI, albumin-bilirubin; AFP, alpha-fetoprotein. Bold values are statistically significant (p<0.05).

The high AFP cohort had an overall response rate (ORR) of 62.5%, including 11 cases (12.5%) achieving CR and 44 cases (50.0%) achieving PR, respectively. In the AFP response subgroup, 9 cases (16.4%) achieved CR, 33 cases (60.0%) achieved PR, resulting in a favorable ORR of 76.4%. In contrast, the ORR of the AFP non-response subgroup was significantly lower at 39.4%, with a statistically significant difference (p=0.001) (**Supplementary Table S2**).

### 3.3 Predictor of objective response in the high-DCP cohort

A total of 91 individuals presented with elevated baseline DCP levels, of whom 66 (72.5%) were over 50 years of age, and the majority were infected with HBV (85, 93.4%). Among this cohort, vascular invasion was observed in 71 patients (78.0%), 17 patients (18.6%) experienced extrahepatic metastases, and the median baseline DCP value was 2386.0 mAU/ml (**Table 1**).

According to ROC curve analysis, a 70% reduction in DCP levels after 6–8 weeks of triple therapy was identified as the optimal cut-off threshold (**Supplementary Table S1**). In this cohort, a total of 42 patients (46.1%) achieved DCP response (**Table 1**). Most patients with a DCP response also obtained objective response, as shown in **Figure 2B**. The multivariate analysis further demonstrated that DCP response independently influenced objective response (OR 7.99; 95% CI 2.82-22.60; p < 0.001), as shown in **Table 3**.

**Table 3:** Univariate and multivariate analyses for factors associated with objective response in the high DCP cohort (n=91).

Variables	Univariate Analyses		Multivariate Analyses	
	OR (95%CI)	p	OR (95%CI)	p
Age, years (≤50/>50)	0.82(0.32-2.10)	0.676		
Gender (Female/Male)	3.88(0.45-33.73)	0.219	3.84(0.39-37.96)	0.250
Extrahepatic spread (No/Yes)	1.02(0.36-2.89)	0.977		
BCLC(B/C)	0.69(0.17-2.78)	0.607		
Child-Pugh (A/ B)	1.47(0.56-3.85)	0.428		
Tumor size (cm) (≤5/>5)	0.68(0.28-1.63)	0.385		
Baseline DCP (ng/ml)	1.00(1.00-1.00)	0.522		
ALBI grade (1/2)	1.07(0.36-3.21)	0.905		
DCP response (Yes/No)	8.00(2.85-22.48)	**<0.001**	7.99(2.82-22.60)	**<0.001**
Vascular invasion (No/Yes)	0.92(0.34-2.51)	0.868		

PFS, progression-free survival; BCLC, Barcelona Clinic Liver Cancer staging; ALBI, albumin-bilirubin; DCP, des-gamma-carboxy prothrombin. Bold values are statistically significant (p<0.05).

The high DCP cohort achieved an ORR of 62.6%, with CR observed in 12 cases (13.2%) and PR in 45 cases (49.5%). In the DCP response subgroup, 10 cases (23.8%) achieved CR, and 26 cases (61.9%) achieved PR, resulting in an increased ORR of 85.7%. In contrast, the ORR of 42.9% in the AFP non-response subgroup was lower with a significant difference between the two groups (p=0.001) (**Supplementary Table S2**).

### 3.4 Association between AFP response and PFS in the high-AFP cohort

**Figure 3:**
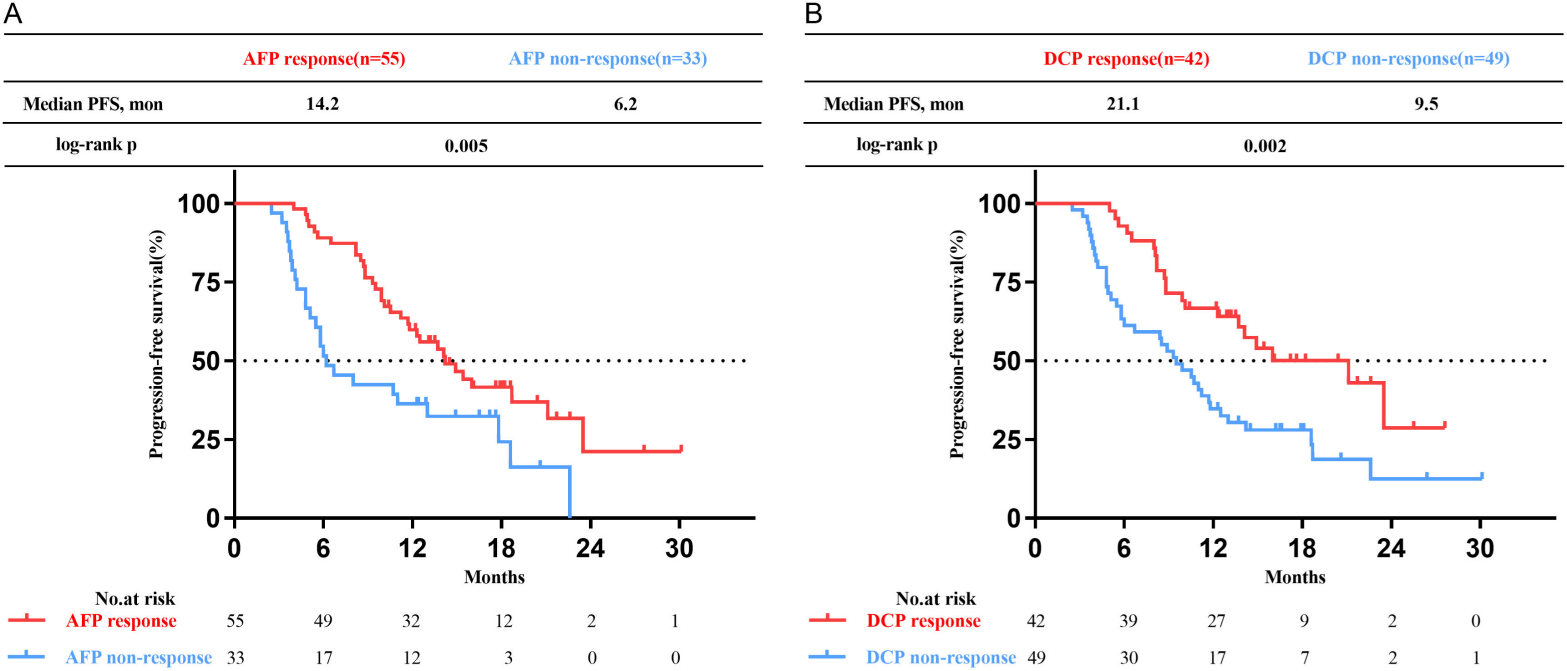
Kaplan-Meier curves of progression-free survival (PFS) for patients with uHCC undergoing triple therapy. **(A)** in the high AFP cohort. **(B)** in the high DCP cohort. AFP, alpha-fetoprotein; DCP, des-gamma-carboxy prothrombin.

The median PFS in high AFP cohort was 11.7 months (95% CI 9.0-14.4 months). Notably, the AFP response subgroup exhibited a longer median PFS compared to those without response (13.7 months vs. 6.2 months, p=0.013), as illustrated in **Figure 3A**. In the multivariate analysis, the absence of extrahepatic metastasis emerged as a favorable and meaningful factor influencing PFS (HR 0.40, 95% CI 0.21-0.75; p=0.004), and achieving AFP response was correlated with increased PFS (HR 0.36; 95% CI 0.20-0.62; p<0.001) (**Table 4**).

**Table 4:** Univariate and multivariate analyses for factors affecting PFS and OS in the high AFP cohort (n=88).

Variables	PFS				OS			
	Univariate Analyses		Multivariate Analyses		Univariate Analyses		Multivariate Analyses	
	HR (95%CI)	p	HR (95%CI)	p	HR (95%CI)	p	HR (95%CI)	p
Age, years (≤50/>50)	0.78(0.44-1.39)	0.400			0.69(0.28-1.70)	0.418		
Gender (Female/Male)	0.46(0.17-1.28)	0.139	0.24(0.08-0.70)	**0.009**	0.78(0.18-3.27)	0.729		
Extrahepatic spread (No/Yes)	0.60(0.34-1.07)	0.084	0.40(0.21-0.75)	**0.004**	0.92(0.39-2.15)	0.846		
BCLC(B/C)	0.74(0.29-1.84)	0.510			0.72(0.17-3.04)	0.658		
Child-Pugh (A/B)	1.06(0.60-1.87)	0.837			0.62(0.29-1.33)	0.218		
Tumor size (cm) (≤5/>5)	1.01(0.61-1.68)	0.973			0.54(0.24-1.21)	0.134		
Baseline AFP (ng/ml)	1.00(1.00-1.00)	0.170			1.00(1.00-1.00)	**0.029**	1.00(1.00-1.00)	0.070
ALBI grade (1/2)	1.05(0.55-2.02)	0.883			0.48(0.15-1.60)	0.234		
AFP response (Yes/No)	0.53(0.32-0.88)	**0.015**	0.36(0.20-0.62)	**<0.001**	0.44(0.21-0.90)	**0.026**	0.47(0.22-0.99)	**0.047**
Vascular invasion (No/Yes)	1.09(0.59-2.01)	0.780			0.54(0.19-1.56)	0.259		

PFS, progression-free survival; OS, overall survival; BCLC, Barcelona Clinic Liver Cancer staging; ALBI, albumin-bilirubin; AFP, alpha-fetoprotein. Bold values are statistically significant (p<0.05).

### 3.5 Association between DCP response and PFS in the high-DCP cohort

The median PFS in the high DCP cohort was 11.2 months (95% CI 8.8-13.5 months), and the DCP response subgroup exhibited a longer median PFS than the DCP non-response subgroup (15.6 months vs. 9.3 months, p=0.001), as shown in **Figure 3B**. In the multivariate analysis, the absence of extrahepatic metastasis was a favorable factor influencing PFS (HR 0.51, 95% CI 0.29-0.93; p=0.027), and achieving DCP response was correlated with increased PFS (HR 0.44, 95% CI 0.26-0.74; p=0.002) (**Table 5**).

**Table 5:** Univariate and multivariate analyses for factors affecting PFS and OS in the high DCP cohort (n=91).

Variables	PFS				OS			
	Univariate Analyses		Multivariate Analyses		Univariate Analyses		Multivariate Analyses	
	HR (95%CI)	p	HR (95%CI)	p	HR (95%CI)	p	HR (95%CI)	p
Age, years (≤50/>50)	0.70(0.39-1.25)	0.228			0.73(0.29-1.72)	0.497		
Gender (Female/Male)	0.50(0.18-1.37)	0.177	0.42(0.15-1.19)	0.102	0.87(0.21-3.66)	0.845		
Extrahepatic spread (No/Yes)	0.56(0.31-0.99)	0.048	0.51(0.29-0.93)	**0.027**	1.17(0.44-3.09)	0.751		
BCLC(B/C)	0.89(0.38-2.07)	0.791			0.71(0.17-2.98)	0.635		
Child-Pugh (A/B)	1.00(0.56-1.77)	0.988			0.67(0.29-1.54)	0.347		
Tumor size (cm) (≤5/>5)	1.28(0.77-2.12)	0.346			0.50(0.20-1.25)	0.137	0.56(0.23-1.40)	0.213
Baseline DCP (ng/ml)	1.00(1.00-1.00)	0.833			1.00(1.00-1.00)	0.413		
ALBI grade (1/2)	0.80(0.41-1.58)	0.529			0.32(0.08-1.34)	0.118		
DCP response (Yes/No)	0.43(0.26-0.73)	**0.002**	0.44(0.26-0.74)	**0.002**	0.33(0.13-0.81)	**0.016**	0.35(0.14-0.86)	**0.022**
Vascular invasion (No/Yes)	1.15(0.64-2.08)	0.644			0.55(0.19-1.59)	0.271		

PFS, progression-free survival; OS, overall survival; BCLC, Barcelona Clinic Liver Cancer staging; ALBI, albumin-bilirubin; DCP, des-gamma-carboxy prothrombin. Bold values are statistically significant (p<0.05).

### 3.6 Prognostic value of the biomarker response for OS

**Figure 4:**
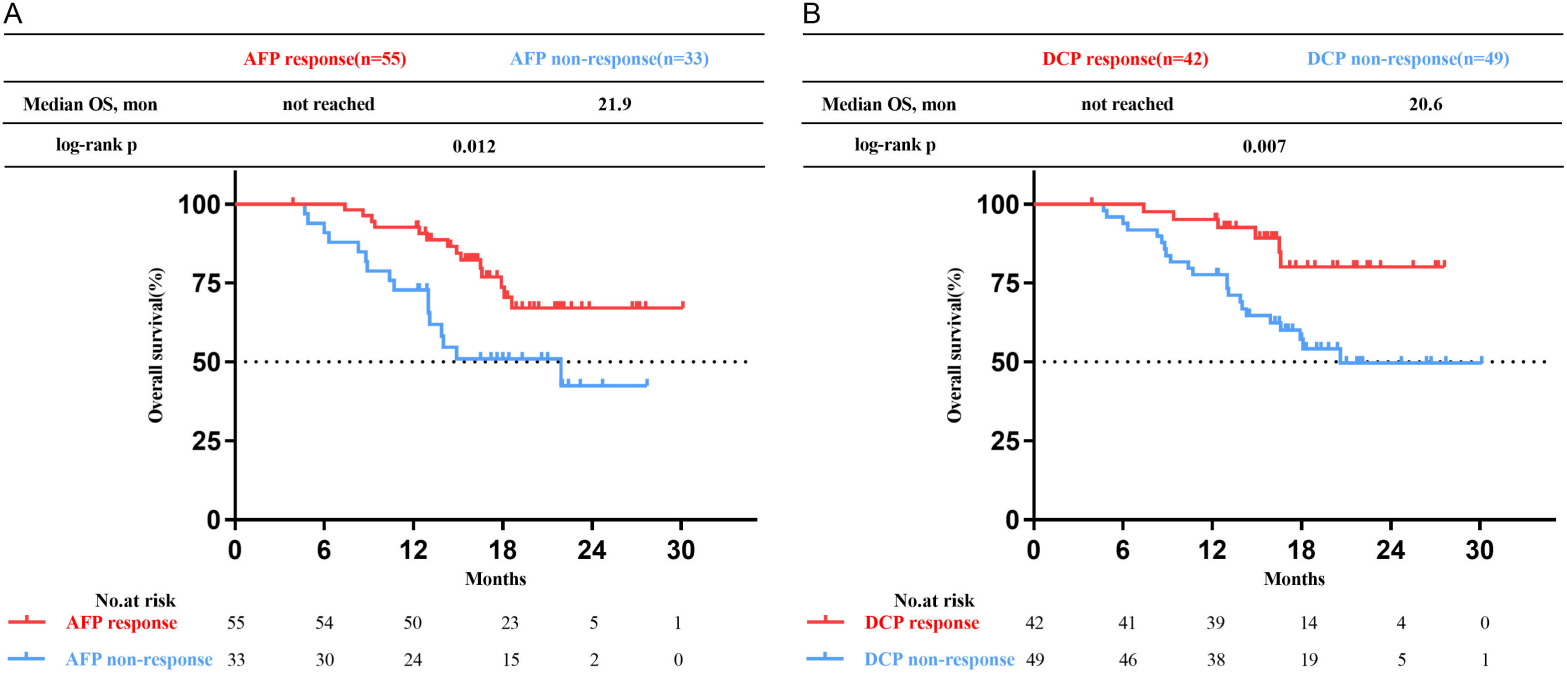
Kaplan-Meier curves of overall survival (OS) for patients with uHCC undergoing triple therapy. **(A)** in the high AFP cohort. **(B)** in the high DCP cohort. AFP, alpha-fetoprotein; DCP, des-gamma-carboxy prothrombin.

Patients who achieved AFP or DCP response exhibited more favorable OS compared to the non-response subgroup (not reached vs. 21.9 months, p=0.012; not reached vs. 20.6 months, p=0.007, respectively) (**Figure 4**). The multivariate analysis revealed that AFP or DCP response was associated with prolonged OS (HR 0.47, 95% CI 0.22-0.99; HR 0.35, 95% CI 0.14-0.86; p=0.047, p=0.022, respectively) (**Tables 4**, **5**).

## 4 Discussion

The triple combination therapy of radiotherapy, anti-angiogenic agents and ICIs has demonstrated a promising improvement in local response and survival outcomes for uHCC (16, 31). Radiotherapy not only induces lethal DNA damage in tumors, but also enhances their antigenicity and adjuvant characteristics, effectively converting cancer cells into *in situ* vaccines and promoting the activation of anticancer immunity (32). Furthermore, ICIs serve to counteract the immunosuppressive effects and amplify abscopal effects of radiotherapy, leading to tumor shrinkage beyond the irradiation field (33, 34). Moreover, anti-angiogenic therapies can improve the effectiveness of radiation therapy by normalizing tumor vasculatures and fostering an immune-friendly tumor microenvironment (35). This research also confirmed the safety and effectiveness of triple treatment, yielding favorable outcomes. However, the current lack of a biomarker capable of accurately identifying individuals likely to benefit from this triple treatment represents a significant unmet clinical need.

Serum AFP, an easily accessible biomarker correlated with tumor burden and biology, has an established role in HCC for decades (36). It is generally recommended for use alongside ultrasound to facilitate early identification of HCC in high-risk individuals (37). Moreover, AFP measurements can be easily repeated during a patient’s follow-up, enabling dynamic assessment of changes to monitor treatment efficacy. Several retrospective researches on systemic therapy for HCC have investigated the prognostic value of AFP as a serum biomarker of response. A recent study found that a drop or rise in AFP over 30% was an independent predictor of objective response and PD, respectively (38). Shao defined early AFP response as a >20% drop from baseline levels at 4 weeks post-therapy, which was linked to increased treatment effectiveness of ICIs for advanced HCC (39). However, few studies have been reported on the prognostic value of AFP for SBRT or triple combination therapy. Additionally, the cutoff defining AFP response was somewhat arbitrary. As far as we are aware, this is the first study to explore the clinical value of tumor biomarker response in patients undergoing SBRT combined with systemic therapy. In the high AFP cohort, we discovered a greater than 50% reduction in AFP values at 6-8 weeks post-therapy independently predicted better OS (HR 0.47, 95% CI 0.22-0.99), PFS (HR 0.39; 95% CI 0.23-0.68) and ORR (OR 5.50, 95% CI 2.04-14.83). The main strength of this study is that the cutoffs defining biomarker responses were derived from ROC curves, adhered to a strict logical foundation. A substantial reduction in AFP levels (>50%) following triple therapy may enhance the confidence of both physicians and patients in choosing this treatment option. It helps in identifying patients who are likely to respond favorably to triple therapy and guide treatment modifications for those who show poor response.

The correlation between DCP and HCC was first reported in 1984 (40). Since then, accumulating evidence has revealed that DCP could serve as an effective diagnostic and prognostic tumor marker for HCC (41). Several research have investigated its usefulness for surveillance, treatment monitoring, and prognosis assessment of HCC (42, 43). Preoperative DCP positivity, but not AFP positivity, was an independent risk factor of early HCC recurrence after hepatectomy (44). DCP monitoring also assists with predicting OS and PFS in TACE (24). Lower pre-treatment DCP was linked to better OS (HR 0.65), and its response post-TACE of ≥20.0-50.0% decrease was associated with improved OS and PFS (HR 0.39 and 0.42, respectively) (25). Unfortunately, the prognostic utility of DCP in HCC patients undergoing SBRT or triple combination therapy remains obscure. In this study, DCP responders, defined as those with a decrease of over 70.0% from baseline, were associated with radiologic response (OR 7.99, 95% CI 2.82-22.60) and had better PFS (HR 0.39, 95% CI 0.23-0.68) and OS (HR 0.35, 95% CI 0.14-0.86) than DCP non-responders. DCP can serve as a valuable indicator for evaluating both immediate and long-term clinical outcomes after triple therapy, especially in patients with elevated baseline DCP levels. This study provides compelling evidence to endorse routine testing of DCP pre- and post- SBRT treatment.

Several limitations that should be acknowledged in this study. Firstly, this study adopted a retrospective design with a modest sample size, thereby introducing an inherent selection bias that cannot be avoided. Secondly, we excluded patients with AFP <10 ng/ml and DCP < 40mAU/ml at baseline, for whom alternate approaches should be considered such as liquid biopsies (45). Thirdly, differences in the types of ICIs and targeted agents may potentially influence the consistency of the treatment process and thus have a slight impact on the conclusions. Lastly, it is worth mentioning that a significant proportion of patients included were diagnosed with hepatitis B-associated HCC, thereby limiting the generality of the findings to a broader population. Despite these acknowledged limitations, we provide supportive rationale for AFP and DCP response cutoffs and tested the prognostic value of them.

## 5 Conclusion

A >50% decrease in AFP or a >70% decrease in DCP, measured 6-8 weeks after triple combination therapy of SBRT, immunotherapy, and targeted therapy for uHCC patients, was associated with improved ORR, PFS and OS. Results from this study demonstrate the clinical value of early biomarker response in predicting the efficacy of SBRT combined with immunotherapy and targeted therapy for patients with uHCC.

## Data Availability

The original contributions presented in the study are included in the article/**Supplementary Material**. Further inquiries can be directed to the corresponding authors.
