# Supplementary material for: Prognostic significance of early alpha fetoprotein and des-gamma carboxy prothrombin responses in unresectable hepatocellular carcinoma patients undergoing triple combination therapy
Source: Front Immunol. 2024 Dec 12;15:1508028. doi: 10.3389/fimmu.2024.1508028 (PMC11669689; doi:10.3389/fimmu.2024.1508028)
Supplement: Supplementary file 1 [file Table1.docx]

**Supplementary Table S1** The cutoff value of reduction in AFP and DCP based on receiver-operation characteristic (ROC) analysis

| Biomarker | AUC | Sensitivity | Specificity | Youden Index | Optimal cutoff, % | Derived cutoff, % |
| --- | --- | --- | --- | --- | --- | --- |
| AFP | 0.702 | 0.737 | 0.777 | 0.404 | 51.5 | 50 |
| DCP | 0.723 | 0.610 | 0.824 | 0.434 | 66.8 | 70 |

Abbreviations: AFP, alpha-fetoprotein; DCP, des-gamma-carboxyprothrombin; AUC, Area under the curve of ROC.

**Supplementary Table S2** Relationship between the radiological response per mRECIST and the response of tumor markers.

| Response | Total(n=95) | High-AFP | | | High-DCP | | |
| --- | --- | --- | --- | --- | --- | --- | --- |
|  |  | Total n=88 | AFP responder  (n=55) | AFP non-responder  (n=33) | Total n=91 | DCP responder  (n=42) | DCP non-responder  (n=49) |
| CR | 14 | 11 | 9 | 2 | 12 | 10 | 2 |
| PR | 47 | 44 | 33 | 11 | 45 | 26 | 19 |
| SD | 13 | 12 | 6 | 6 | 13 | 5 | 8 |
| PD | 21 | 21 | 7 | 14 | 21 | 1 | 20 |
| ORR (%) | 64.2 | 62.5 | 76.4 | 39.4 | 62.6 | 85.7 | 42.9 |
| P value |  |  | 0.001 | |  | 0.001 | |

Abbreviations: mRECIST, modified response evaluation criteria in solid tumors; CR, complete response; PR, partial response; SD, stable disease; PD, progressive disease; ORR, objective response rate; AFP, alpha-fetoprotein; DCP, des-gamma-carboxy prothrombin.
